# Therapy-related myeloid neoplasms following treatment for high-risk gestational trophoblastic neoplasia: a case series and retrospective analysis

**DOI:** 10.1007/s10147-026-03073-4

**Published:** 2026-06-04

**Authors:** Kaoru Niimi, Eiko Yamamoto, Kosuke Yoshida, Yuko Yasui, Hiroaki Kajiyama

**Affiliations:** 1 Department of Obstetrics and Gynecology, Nagoya University Graduate School of Medicine, 65 Tsurumai-Cho, Showa-Ku, Nagoya, 466-8550 Japan; 2 Department of Healthcare Administration, Nagoya University Graduate School of Medicine, Nagoya, Japan

**Keywords:** Choriocarcinoma, Therapy-related myeloid neoplasm, Secondary neoplasm

## Abstract

**Background:**

Therapy-related myeloid neoplasms (t-MNs), including therapy-related myelodysplastic syndrome and therapy-related acute myeloid leukemia, are rare but severe late complications of cytotoxic chemotherapy. This study aimed to identify the occurrence of t-MNs in patients with high-risk gestational trophoblastic neoplasia (GTN) treated at our institution.

**Methods:**

A retrospective review was conducted in 47 patients with high-risk GTN treated between 1990 and 2023. Clinical data included age, antecedent pregnancy, International Federation of Gynecology and Obstetrics stage, World Health Organization prognostic score, chemotherapy regimen, cumulative etoposide dose, and outcomes. Patients who developed t-MN were compared with those who did not.

**Results:**

Among the 47 patients, 34 (72%) achieved cure without relapse, 6 (13%) achieved cure following relapse, and 7 (15%) did not achieve cure. Four patients (8.5%) developed t-MN after prolonged chemotherapy. The latency from initiation of GTN treatment to t-MN onset ranged from 3 to 5 years, whereas the interval from the last chemotherapy administration to diagnosis ranged from immediately after treatment completion to 20 months. All four patients received multi-agent, etoposide-containing regimens with cumulative doses ≥ 6,000 mg/m^2^. Cytogenetic and molecular analyses revealed KMT2A (formerly MLL) rearrangements and IDH1/2 mutations. Despite allogeneic hematopoietic stem cell transplantation, clinical outcomes were unfavorable, and all patients ultimately died of t-MN or associated complications.

**Conclusion:**

Although combination chemotherapy remains essential for high-risk GTN, exposure to high cumulative doses of etoposide confers a risk of secondary t-MNs. Long-term hematologic surveillance and less leukemogenic strategies are warranted.

## Introduction

Therapy-related myeloid neoplasms (t-MNs), including therapy-related myelodysplastic syndrome (t-MDS) and acute myeloid leukemia (t-AML), are increasingly recognized as severe late complications of cytotoxic therapy. These disorders are associated with poor prognoses, with most studies reporting a median survival of less than 2 years [1–4]. Among the cytotoxic agents, topoisomerase II inhibitors—such as etoposide—have been strongly implicated in leukemogenesis, particularly in patients harboring rearrangements of the KMT2A (formerly MLL) gene on chromosome 11q23 [5]. Etoposide is a key component of most standard and salvage regimens for high-risk GTN, often administered repeatedly over prolonged treatment courses, making cumulative exposure a clinically relevant and potentially modifiable risk factor for secondary t-MN [6].

Gestational trophoblastic neoplasia (GTN) is highly chemosensitive, and the prognosis of high-risk disease has improved substantially with the use of combination chemotherapy. However, patients with metastatic or refractory choriocarcinoma often require multiple cycles of intensive multi-agent regimens, many of which include etoposide. Although these treatments have improved survival rates, long-term survivors remain at risk of developing late treatment-related toxicities. Previous studies have reported secondary malignancies in 2%–4.5% of patients with GTN, with a disproportionately increased incidence of acute leukemia compared with the general population, particularly among those treated with combination chemotherapy [6, 7]. However, most of these studies were limited by small numbers of therapy-related myeloid neoplasms, heterogeneous treatment regimens, and relatively short or variable follow-up durations, restricting detailed evaluation of treatment exposure and clinical outcomes.

Nevertheless, detailed descriptions of t-MNs arising after GTN remain limited, particularly regarding the patterns of chemotherapy exposure, clinical course, and outcomes. To address this knowledge gap, the occurrence of t-MN was examined in a cohort of patients with high-risk GTN treated at our institution, with a particular focus on cumulative etoposide exposure and long-term clinical outcomes. The findings aim to guide risk stratification and post-treatment surveillance.

## Materials and methods

### Study design and patients

A retrospective cohort study was performed in patients with high-risk GTN treated at Nagoya University Hospital between January 1990 and December 2023. High-risk GTN was defined according to the International Federation of Gynecology and Obstetrics (FIGO) 2000 risk scoring system (score ≥ 7). A total of 47 patients were identified from institutional medical records, of whom four subsequently developed t-MNs, including t-MDS and t-AML.

### Data collection

Clinical data were retrospectively obtained from patient charts, operative records, pathology reports, and institutional chemotherapy databases. Collected information included patient demographics, such as age and antecedent pregnancy; clinical characteristics, including FIGO stage and World Health Organization (WHO) prognostic score; treatment details, such as the number of chemotherapy cycles, type of regimen, and cumulative drug exposure, with a particular focus on etoposide; laboratory data, including serum human chorionic gonadotropin (hCG) levels at treatment initiation; and survival outcomes.

### Ethical approval

This study was conducted in accordance with the Declaration of Helsinki and approved by the Institutional Review Board of the Nagoya University Graduate School of Medicine (approval no. 2019-0106). The requirement for written informed consent was waived due to the retrospective nature of the study, and an opt-out method was provided on the institutional website.

### Statistical analysis

Comparisons between patients with and without t-MNs were performed using the Mann–Whitney U test for continuous variables. Statistical significance was defined as a two-sided p-value of < 0.05. All statistical analyses were conducted using GraphPad Prism version 10.6.1 (GraphPad Software, San Diego, CA, USA).

## Results

A total of 47 patients with high-risk GTN were included in this study. The clinical characteristics of the study population are summarized in Table 1. The median age at diagnosis was 38 years (range: 17–70 years). Antecedent pregnancies included normal deliveries, abortions, and hydatidiform moles. All patients met the FIGO criteria for high-risk disease (score ≥ 7), with the WHO prognostic scores demonstrating a broad distribution across the cohort. The median serum hCG concentration at treatment initiation was 22,650.55 IU/L (range, 4.5–1,900,000 IU/L). All patients received multiagent chemotherapy. The most frequently administered initial regimens were MAC, MEA, and EMA/CO, with a median of 13 chemotherapy courses delivered per patient (range, 2–55 courses).

**Table 1:** Clinical characteristics of 47 patients with high-risk GTN

Variable	n (%) or median [range]
Age at diagnosis (y)	38 [17–70]
FIGO stage	I: 11 (23%)
	II: 0
	III: 18 (38%)
	IV: 18 (38%)
WHO score categories	7–12: 31 (66%)
	≥ 13: 16 (34%)
hCG at treatment initiation	22,650.55 IU/L [4.5–1,900,000]
Number of chemotherapy courses	13 [2–55]
Cumulative etoposide dose category (mg/m^2^)	< 1,500 mg/m^2^: 3 (6%)
	1,500–2,999: 21 (45%)
	≥ 3,000: 22 (47%)
Outcome	Cured: 34 (72%)
	Cured after relapse: 6 (13%)
	Died: 7 (15%)
Therapy-related myeloid neoplasms (t-MNs)	4 (8.5%)

FIGO, International Federation of Gynecology and Obstetrics; hCG, human chorionic gonadotropin; WHO, World Health Organization

The median cumulative etoposide dose for the entire cohort was 2800 mg/m^2^ (range, 0–15,653 mg/m^2^). When stratified by cumulative exposure, 3 patients (6%) received < 1500 mg/m^2^, 21 (45%) received 1500–2999 mg/m^2^, and 22 (47%) received ≥ 3000 mg/m^2^. In terms of clinical outcomes, 34 patients (72%) achieved complete remission without relapse. Six patients (13%) experienced disease relapse but were successfully cured; this group included three patients who subsequently died of t-MNs. Seven patients (15%) failed to achieve cure and eventually died due to GTN. Overall, four patients (8.5%) developed t-MN following GTN treatment. This occurrence should be interpreted in the context of our tertiary referral setting, which includes a high proportion of heavily pretreated and refractory cases. The latency period from GTN treatment initiation to the diagnosis of t-MNs ranged from 3 to 5 years among the four affected patients. When calculated from the completion of the final chemotherapy cycle, the interval to t-MN onset varied widely, ranging from immediately after treatment completion to 20 months. The detailed clinical courses of these four patients are presented in Table 3.

The clinical characteristics were compared between patients with and without t-MNs (Table 2). No significant difference was observed in age at diagnosis between the two groups (median, 41.5 vs. 37 years; *p* = 0.83). Similarly, the WHO prognostic score tended to be higher in the t-MN group (median: 14 vs. 10), although this difference was not significant (*p* = 0.095).

**Table 2:** Comparison of clinical and treatment characteristics between patients with and without t-MNs

Variables	With t-MNs (n = 4)	Without t-MNs (n = 43)	*p*
Age (y)	41.5	37	0.83
WHO scores	14	10	0.095
Cumulative etoposide dose (mg/m^2^)	7,817	2,667	0.0059
Chemotherapy courses	51	13	0.0024

Data are shown as medians. t-MN, therapy-related myeloid neoplasms; WHO, World Health Organization

**Fig. 1:**
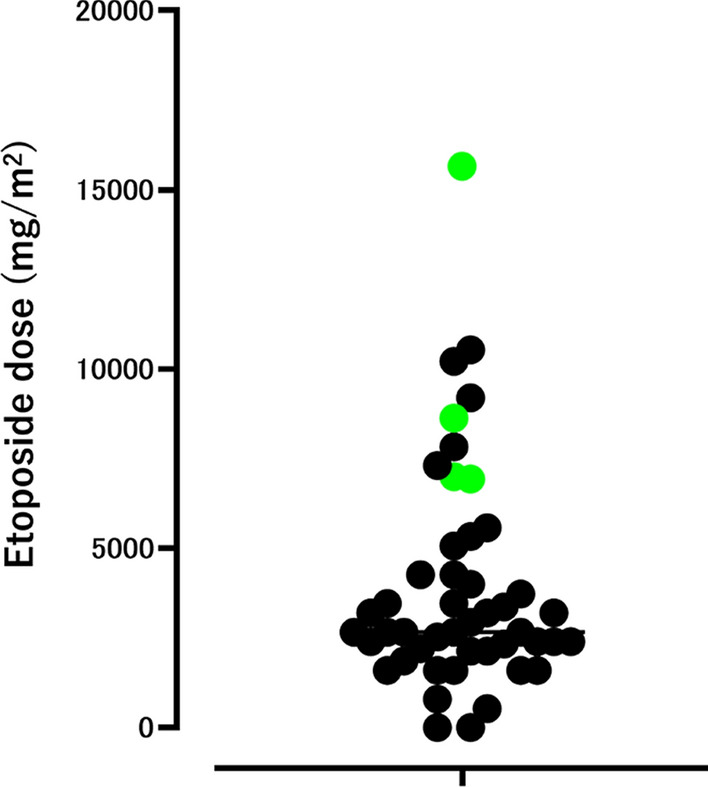
Individual cumulative etoposide dose in patients with high-risk GTN. Green dots denote patients who developed t-MN, whereas black dots denote those who did not. GTN, gestational trophoblastic neoplasia

**Fig. 2:**
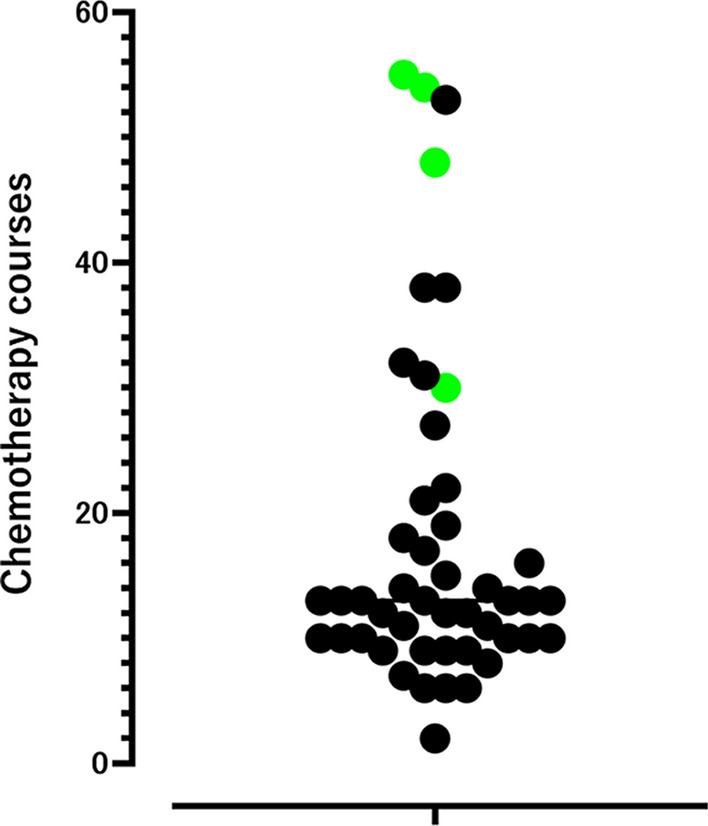
Numbers of chemotherapy courses administered to patients with high-risk GTN. Green dots denote patients who developed t-MN, whereas black dots denote those who did not. GTN, gestational trophoblastic neoplasia

By contrast, both cumulative etoposide exposure and number of chemotherapy courses were significantly greater among patients who developed t-MNs. The median cumulative etoposide dose was 7,817 mg/m^2^ in the t-MN group compared with 2,667 mg/m^2^ in the non-t-MN group (*p* = 0.0059). Similarly, the median numbers of chemotherapy courses were 51 and 13 in the t-MN and non-t-MN groups, respectively (*p* = 0.0024). Patients who developed t-MNs tended to have been exposed more often to cumulative amounts of etoposide and more courses of chemotherapy (Figs. 1 and 2). The number of chemotherapy courses may also serve as a surrogate marker of overall treatment burden. However, the groups overlapped, indicating that prolonged treatment did not uniformly result in t-MN development. These findings indicated that prolonged chemotherapy and cumulative etoposide are important but not sufficient risk factors for the development of t-MNs in patients with high-risk GTN.

A detailed description of the four patients who developed t-MNs is provided in the following section, with treatment exposure and additional clinical characteristics summarized in Table 3 and 4.

**Table 3:** Chemotherapy exposure before onset of therapy-related myeloid neoplasms

Patient 1		Patient 2		Patient 3		Patient 4	
Regimen	Courses	Regimen	Courses	Regimen	Courses	Regimen	Courses
EMA-CO	5	MTX	2	MTX	4	MTX	7
EP	2	MAC	2	ACTD	3	EMA-CO	10
5-FU/CDDP/ETP	1	EMA-CO	2	MEA	3	EPEMA	15
EP	2	EA	7	EMA-CO	8	FA	3
EA	7	EA	9	EPEMA	5	TCTE	3
EP	2	MEA	8	MTX	4	TCTE	2
EA	3			EA	1	MEA	7
TC	2			MEA	1	MEA	1
EA/EP	4			EP/EMA	5		
mini ICE	8			TPTE	2		
mini ICE	8			Pembrolizumab	10		
CPT-11/ETP	3			TCTE	8		
EA	5						
CPA/ETP/CDDP	3						
Total	55	Total	30	Total	54	Total	48
Total etoposide (mg/m^2^)	18,920	Total etoposide (mg/m^2^)	7,000	Total etoposide (mg/m^2^)	6,930	Total etoposide (mg/m^2^)	8,633

ACTD, actinomycin D; CDDP, cisplatin; CPA, cyclophosphamide; CPT-11, irinotecan; EA, etoposide and actinomycin D; EMA-CO, etoposide, methotrexate, actinomycin D, cyclophosphamide, and vincristine (Oncovin); EP, etoposide and cisplatin; ETP, etoposide; MAC, methotrexate, actinomycin D, and cyclophosphamide; EPEMA, etoposide, cisplatin, etoposide, methotrexate, and actinomycin D; FA, floxuridine and actinomycin D; ICE, ifosfamide, carboplatin, and etoposide; MEA, methotrexate, etoposide, and actinomycin D; mini ICE, dose-reduced ifosfamide, carboplatin, and etoposide; MTX, methotrexate; TC, paclitaxel and carboplatin; TCTE, paclitaxel, carboplatin, paclitaxel, and etoposide; TPTE, paclitaxel, cisplatin, paclitaxel, and etoposide

**Table 4:** Clinical summary of four patients with therapy-related myeloid neoplasms

Patient No	Age (y)	FIGO stage/score	hCG (mIU/mL)	Time to remission (months)	Interval to t-MN (months)	t-MN subtype	Cytogenetic/molecular findings	Survival after t-MN
1	28	IV:15	360,000	45	20	t-AML	45,XX,t(11;19)(q23;p13), add(12)(p11)	6 m after t-AML
2	45	I:11	1,406	30	6	t-MDS → AML	Normal karyotype	8 y after t-MDS
3	39	IV:18	125,000	38	18	t-MDS → AML	IDH1 and IDH2 mutations	31 m after t-MDS
4	47	III:13	82,186	32	During treatment (No remission)	t-MDS → AML		2 m after t-MDS

AML, acute myeloid leukemia; FIGO, International Federation of Gynecology and Obstetrics; hCG, human chorionic gonadotropin; HSCT, hematopoietic stem cell transplantation; m, months; MDS, myelodysplastic syndrome; t-MN, therapy-related myeloid neoplasm; y, years

### Patient 1

A 28-year-old Japanese woman, gravida 2, para 1, with a history of a previous one-molar pregnancy, developed high-risk GTN 11 months after a term delivery. At diagnosis, lung and liver metastases were observed. Histopathological examination performed following partial pneumonectomy during chemotherapy confirmed the diagnosis of choriocarcinoma.

The patient received 55 courses of various chemotherapy regimens and ultimately achieved complete remission. The cumulative doses of key chemotherapeutic agents were 18,920 mg/m^2^ for etoposide, 1,080 mg/m^2^ for cisplatin, and 4,800 mg/m^2^ for cyclophosphamide.

Twenty months after completion of chemotherapy, the patient presented to a general practitioner with complaints of fatigue. Laboratory evaluation revealed refractory anemia and circulating blast cells. Bone marrow aspiration demonstrated > 40% blasts expressing CD33, consistent with AML, classified as French–American–British (FAB) subtype M0. Cytogenetic analysis revealed an abnormal female karyotype, 45,XX, t(11;19)(q23;p13), add(12)(p11), findings consistent with therapy-related AML secondary to etoposide exposure.

Induction chemotherapy for AML was administered, followed by allogeneic hematopoietic stem cell transplantation; however, graft failure occurred. Despite subsequent supportive care, the patient died 6 months after the diagnosis of AML.

### Patient 2

A 45-year-old Japanese woman, gravida 4, para 2, with a history of one abortion and one molar pregnancy, was treated for low-risk GTN 8 years earlier. She received 13 courses of chemotherapy for hydatidiform mole-associated GTN and achieved complete remission. Four years later, the patient delivered a healthy infant.

More recently, the patient underwent emergency laparotomy for hemoperitoneum, during which a uterine tumor was resected and pathologically diagnosed as choriocarcinoma. Seventeen courses of multidrug chemotherapy were administered, resulting in remission. However, 15 weeks after the last chemotherapy cycle, uterine recurrence of choriocarcinoma was detected, and a total abdominal hysterectomy was performed. Following surgery, serum hCG levels spontaneously declined to below the cutoff value, and remission was achieved. The cumulative doses of etoposide and cyclophosphamide were 7,000 and 2,480 mg/m^2^, respectively.

Several months after surgery, multiple papular skin lesions developed on the trunk. Histopathological examination of a skin biopsy revealed myeloid sarcoma. Bone marrow aspiration demonstrated 2% blast cells, leading to the diagnosis of MDS. Cytogenetic analysis revealed a normal female karyotype. Conservative management for MDS was pursued for approximately 2 years.

Subsequently, granulocytic sarcoma developed, and the patient was treated with systemic chemotherapy, followed by allogeneic bone marrow transplantation. Although remission was initially achieved, leukemia recurred 6 months later, presenting with subcutaneous nodules. A cauda equina mass subsequently developed, necessitating whole-brain and whole-spinal cord irradiation.

One year later, chemotherapy was administered for leukemic infiltration of the stomach. Despite treatment, the disease progressed with involvement of the periventricular region. The patient’s level of consciousness gradually deteriorated, and death ultimately ensued.

### Patient 3

A 39-year-old Japanese woman, gravida 1, with a history of one abortion developed high-risk GTN with pulmonary metastases 11 months after the abortion. Total hysterectomy was performed, followed by 54 courses of multidrug chemotherapy, including pembrolizumab, resulting in complete remission. The remission course of this patient has been described previously [8]; therefore, only features pertinent to the development of t-MNs are summarized herein. The cumulative doses of the principal agents administered were 6,930 mg/m^2^ for etoposide, 870 mg/m^2^ for cisplatin (equivalent to 2,040 mg/kg body weight for carboplatin), and 3,000 mg/m^2^ for cyclophosphamide.

Eighteen months following the completion of chemotherapy, t-MDS was diagnosed based on bone marrow aspiration, which revealed 13.8% blast cells. Despite treatment, the disease progressed to AML with hematopoietic lineage abnormalities. Genetic testing revealed mutations in the IDH1 and IDH2 genes. Chemotherapy followed by allogeneic hematopoietic stem cell transplantation was administered, resulting in complete remission.

However, 18 months later, the patient was hospitalized for pneumonia. One month later, she died of pulmonary mucormycosis, which developed secondary to graft-versus-host disease and profound treatment-related immunosuppression.

### Patient 4

A 47-year-old Japanese woman, gravida 4, para 2, with a history of one molar pregnancy initially received seven courses of methotrexate for low-risk GTN following a hydatidiform mole and achieved remission. Two years and eight months later, she developed high-risk GTN with multiple pulmonary metastases. Pathological examination of the uterus following hysterectomy confirmed choriocarcinoma. Despite 41 courses of multidrug chemotherapy, remission was not achieved. The cumulative doses of etoposide and cisplatin (carboplatin) were 8,633 and 1,125 mg/m^2^ (230 mg/body), respectively.

During treatment for septic shock following the last chemotherapy session, blast cells and thrombocytopenia were detected in the peripheral blood, raising the suspicion of t-MDS. Pembrolizumab was administered; however, 1 week later, the patient developed intracranial hemorrhage secondary to metastatic disease, necessitating emergency craniotomy. Bone marrow aspiration at that time revealed 25.5% blast cells, consistent with the transformation to AML.

Profound thrombocytopenia (platelet count 3,000/μL) rendered bleeding uncontrollable. Despite supportive care, the patient died 50 days after receiving pembrolizumab treatment.

## Discussion

In this retrospective cohort of patients with high-risk GTN, t-MNs developed in 4 of 47 patients (8.5%) following intensive multi-agent chemotherapy. All affected patients had received prolonged etoposide-containing regimens, highlighting a potential leukemogenic risk associated with high cumulative cytotoxic exposure to cytotoxic agents in this population. These findings underscore the need to balance curative treatment intensity with the risk of late hematologic toxicity in highly chemosensitive malignancies such as GTN.

Therapy-related MNs, including t-MDS and t-AML, are increasingly recognized as severe late complications of cytotoxic chemotherapy and/or radiotherapy, accounting for approximately 7–8% of all AML cases. In the most recent WHO 5th edition and International Consensus Classification 2022, t-MNs are no longer classified as a distinct disease category but are designated as myeloid neoplasms with a “therapy-related” qualifier, reflecting their distinct pathogenesis and generally poor prognosis [9–11]. Despite advances in supportive care and transplantation, outcomes remain dismal, with median survival typically less than 1 year and 5-year survival rates below 10% in most series [12]. Among patients with t-AML and t-MDS who underwent allogeneic hematopoietic stem cell transplantation, the median overall survival was approximately 19–20 months for both t-AML and t-MDS. Long-term survival is largely restricted to transplant recipients, underscoring the curative potential of alloSCT in this high-risk population [13], particularly in the absence of high-risk cytogenetic abnormalities, such as monosomy 5/7, complex karyotypes, or TP53 mutations [14, 15].

In GTN, secondary malignancies have been reported as late adverse events associated with combination chemotherapy. In a large cohort of 818 patients with GTN, Sisti et al. identified a 2.3% incidence of secondary malignancies, with acute leukemia and thyroid cancer being the most common [7]. Similarly, Savage et al. found that 4.5% of 1,903 GTN survivors developed subsequent malignancies, with increased risks of oral cancer, melanoma, meningioma, and leukemia; notably, the excess risk of leukemia was confined to patients who had undergone multi-agent chemotherapy [6]. Compared with these population-based findings, the relatively high incidence of t-MN in our cohort likely reflects referral bias and treatment intensity. Our institution is a tertiary referral center for refractory and heavily pretreated, high-risk GTN, where patients often undergo prolonged multi-agent chemotherapy. Therefore, the absolute incidence described herein should not be interpreted as being representative of all patients with high-risk GTN. Rather, our findings are most generalizable to patients with refractory or heavily pretreated disease who require further regimens containing etoposide. In contrast, patients whose standard first-line therapy was successful, might have substantially lower risks.

Our series documents four patients who developed t-MNs following intensive treatment for GTN. All affected patients had received prolonged multi-agent chemotherapy, with cumulative etoposide exposure ≥ 6,000 mg/m^2^, supporting a dose-dependent leukemogenic risk. Rather than representing a strict threshold, this level of cumulative exposure may be considered a “high-risk zone” for leukemogenic potential. These results were consistent with the findings of other highly chemosensitive malignancies, particularly germ cell tumors, in which cumulative etoposide exposure is associated with therapy-related leukemia. The risk of secondary AML might increase in cohorts with germ cell tumors treated with cumulative etoposide doses exceeding ~ 2,000 mg/m^2^, although the overall incidence remains relatively low in curative treatment settings [16]. Similarly, epidemiologic analyses among various malignancies have found that therapy-related myeloid neoplasms are strongly linked to treatment intensity and cumulative exposure to topoisomerase II inhibitors such as etoposide. However, in our cohort, many patients with etoposide exposure exceeding 2,000 mg/m^2^ did not develop t-MNs (Fig. 1), underscoring the substantial interindividual variability in susceptibility and suggesting that additional host-related or treatment-related factors contribute to leukemogenesis. Etoposide remains a cornerstone of therapy for high-risk GTN. However, evidence does not justify restricting its use solely owing to the risk of t-MN. Premature discontinuation of effective therapy due to concerns about secondary malignancies might compromise the likelihood of achieving remission. Rather, these results support a risk-adapted approach in which cumulative exposure is documented and balanced against disease control.

Several additional aspects of our study merit emphasis. The interval from the final chemotherapy cycle to the diagnosis of t-MNs ranged from immediately after treatment completion to 20 months. These relatively short latencies are consistent with etoposide-induced leukemogenesis, which typically manifests within 1–3 years [5]. Cytogenetic and molecular abnormalities characteristic of therapy-related diseases, including KMT2A rearrangements (t[11, 19]) and IDH1/2 mutations, were also identified. Although allogeneic transplantation has been attempted in some patients, outcomes remained poor due to transplant failure, relapse, or infectious complications such as mucormycosis, highlighting both the aggressiveness of t-MNs and the considerable treatment challenges faced by heavily pretreated GTN survivors. These findings reinforce the importance of early recognition of cytopenias and timely hematologic referral.

This study has some limitations. The sample size was small, limiting statistical power. Accordingly, the statistical comparisons should be interpreted with caution given the small sample size and limited number of events. As a tertiary referral center, our institution likely overrepresents refractory and metastatic disease, contributing to lower survival rates compared with population-based series. This retrospective design introduces the potential for incomplete or biased data capture, particularly regarding treatment histories from outside institutions. In addition, the long study period may have influenced outcomes due to changes in diagnostic methods, chemotherapy regimens, and supportive care. In particular, temporal changes in treatment strategies may have affected cumulative exposure patterns and survivorship outcomes. Nevertheless, the detailed longitudinal treatment data provide unique insight into cumulative exposure profiles that are rarely captured in population-based studies. In addition, competing risks, including death from GTN or treatment-related complications, may have influenced the observed incidence of t-MNs.

From a reproducibility standpoint, the key variables of cumulative etoposide dose, total chemotherapy courses, treatment duration, and latency to hematologic abnormalities identified herein are routinely available in clinical practice and can be evaluated in external cohorts. This provides a pragmatic framework for future multicenter validation and risk stratification efforts.

When integrated with international evidence in GTN and other highly chemosensitive malignancies, our findings support a model in which leukemogenic risk reflects the cumulative treatment burden rather than single-drug exposure alone, with substantial variability among hosts. This highlights the need for collaborative datasets that can incorporate genetic susceptibility, treatment intensity, and survivorship outcomes.

Our data support the continued clinical application of regimens containing etoposide with intent to cure high-risk GTN, while emphasizing structured long-term hematologic surveillance in survivors who have been heavily treated. Such surveillance may include periodic blood counts and clinical assessment for early detection of hematologic abnormalities. Future directions include multicenter registry studies, refinement of dose-exposure thresholds, and the development of treatment strategies that preserve curative efficacy while mitigating late hematologic toxicity.

## Conclusions

In conclusion, our findings demonstrate the rare but clinically significant development of t-MNs following GTN treatment. Etoposide-based combination chemotherapy remains essential for curing high-risk GTN; however, heightened vigilance for leukemogenic risks is essential, particularly with high cumulative doses. These findings are most relevant to heavily pretreated, high-risk populations, which represent a distinct clinical subgroup within the broader spectrum of GTN, and should be interpreted in the context of treatment intensity rather than generalized to all patients with GTN. Long-term hematologic monitoring and strategies to reduce cytotoxic exposure such as the development of safer regimens or the incorporation of targeted agents are warranted while maintaining the high cure rates achieved with current GTN therapy. Future multicenter studies are needed to refine risk thresholds and establish practical surveillance strategies for survivors.

## Data Availability

The datasets generated and/or analyzed during the current study are not publicly available due to patient privacy and confidentiality concerns, but are available from the corresponding author on reasonable request.

## Abbreviations

AML: Acute myeloid leukemia
alloSCT: Allogeneic hematopoietic stem cell transplantation
CI: Confidence interval
EMA/CO: Etoposide, methotrexate, actinomycin D/cyclophosphamide, vincristine
FIGO: International Federation of Gynecology and Obstetrics
FAB: French–American–British
GTN: Gestational trophoblastic neoplasia
hCG: Human chorionic gonadotropin
IDH: Isocitrate dehydrogenase
MAC: Methotrexate, actinomycin D, cyclophosphamide
MEA: Methotrexate, etoposide, actinomycin D
MDS: Myelodysplastic syndrome
t-MN: Therapy-related myeloid neoplasm
t-MDS: Therapy-related myelodysplastic syndrome
t-AML: Therapy-related acute myeloid leukemia
WHO: World Health Organization
